# Prospective Comparison of Medical Oncologists and a Machine Learning Model to Predict 3-Month Mortality in Patients With Metastatic Solid Tumors

**DOI:** 10.1001/jamanetworkopen.2022.14514

**Published:** 2022-05-31

**Authors:** Finly J. Zachariah, Lorenzo A. Rossi, Laura M. Roberts, Linda D. Bosserman

**Affiliations:** 1 Department of Supportive Care Medicine, City of Hope National Medical Center, Duarte, California; 2 Department of Applied AI and Data Science, City of Hope National Medical Center, Duarte, California; 3 Department of Clinical Informatics, City of Hope National Medical Center, Duarte, California; 4 Department of Medical Oncology, City of Hope National Medical Center, Duarte, California

## Abstract

**Importance:**

To date, oncologist and model prognostic performance have been assessed independently and mostly retrospectively; however, how model prognostic performance compares with oncologist prognostic performance prospectively remains unknown.

**Objective:**

To compare oncologist performance with a model in predicting 3-month mortality for patients with metastatic solid tumors in an outpatient setting.

**Design, Setting, and Participants:**

This prognostic study evaluated prospective predictions for a cohort of patients with metastatic solid tumors seen in outpatient oncology clinics at a National Cancer Institute–designated cancer center and associated satellites between December 6, 2019, and August 6, 2021. Oncologists (57 physicians and 17 advanced practice clinicians) answered a 3-month surprise question (3MSQ) within clinical pathways. A model was trained with electronic health record data from January 1, 2013, to April 24, 2019, to identify patients at high risk of 3-month mortality and deployed silently in October 2019. Analysis was limited to oncologist prognostications with a model prediction within the preceding 30 days.

**Exposures:**

Three-month surprise question and gradient-boosting binary classifier.

**Main Outcomes and Measures:**

The primary outcome was performance comparison between oncologists and the model to predict 3-month mortality. The primary performance metric was the positive predictive value (PPV) at the sensitivity achieved by the medical oncologists with their 3MSQ answers.

**Results:**

A total of 74 oncologists answered 3099 3MSQs for 2041 patients with advanced cancer (median age, 62.6 [range, 18-96] years; 1271 women [62.3%]). In this cohort with a 15% prevalence of 3-month mortality and 30% sensitivity for both oncologists and the model, the PPV of oncologists was 34.8% (95% CI, 30.1%-39.5%) and the PPV of the model was 60.0% (95% CI, 53.6%-66.3%). Area under the receiver operating characteristic curve for the model was 81.2% (95% CI, 79.1%-83.3%). The model significantly outperformed the oncologists in short-term mortality.

**Conclusions and Relevance:**

In this prognostic study, the model outperformed oncologists overall and within the breast and gastrointestinal cancer cohorts in predicting 3-month mortality for patients with advanced cancer. These findings suggest that further studies may be useful to examine how model predictions could improve oncologists’ prognostic confidence and patient-centered goal-concordant care at the end of life.

## Key Points

**Question:**

How do oncologists and a machine learning model compare in predicting 3-month mortality for patients with advanced solid tumors?

**Findings:**

In this prognostic study, the machine learning model significantly outperformed 74 oncologists in predicting 3-month mortality for 2041 patients with metastatic solid tumors overall and in gastrointestinal and breast cancer subpopulations. Findings were not significant in genitourinary, lung, and rare cancer groups.

**Meaning:**

The results of this study suggest the potential for a machine learning model trained with electronic health record data to support oncologists in prognostication and clinical decision-making to improve end-of-life care.

## Teaser

This prognostic study compares the outcomes of oncologists vs a machine learning model in predicting mortality in patients with advanced metastatic cancer.

## Introduction

Patients and families rely on clinicians to provide transparent and precise prognostic information to make informed, value-based choices about end-of-life care.^1,2,3,4,5^ Studies have shown that physicians often overestimate survival or are reticent to discuss prognosis and end-of-life preferences owing to perceived patient distress, rapidly progressive science, and lack of prognostic confidence.^6,7,8,9,10,11,12,13,14,15,16^ This overestimation may result in unwanted care and overuse of health care services near the end of life as evidenced by findings that most patients die outside the home and patient preferences are followed completely only about half of the time.^17,18,19^ Within the oncology population, there remains high use of intensive care and chemotherapy and underuse of hospice care near the end of life, costing billions of dollars to the US health care system.^20,21^ Improving prognostic confidence and facilitating alignment between patient values and therapeutic delivery represents an important value proposition for patients, caregivers, clinicians, and payers.^22^

Reliable and consistently applied prognostic tools in oncology may enhance prognostic confidence, increase prognostic authority, and improve the clarity and strength of medical recommendations for and against therapies.^23^ Many prognostic scales have been studied in oncology, some with more administrative burden.^9,10^ A widely and easily implemented prognostic tool is the surprise question (SQ), which asks clinicians whether it would surprise them if a patient died within a particular time frame. The SQ has been used most commonly with a 1-year time frame but also with time frames between 1 week and 6 months with varying performance.^7,9,10,24,25,26,27,28,29,30,31,32,33,34,35,36,37,38,39,40,41^ The SQ has performed better in oncology populations compared with heart failure, kidney failure, and all diagnoses examined in other studies,^7,9,10^ albeit modestly, and further research in this area is needed.

Institutions have increasingly used machine learning (ML) to identify patients at high risk of mortality at different points from 30 days to 5 years for activating care teams to conduct goals-of-care discussions and engage palliative care.^42,43,44,45,46,47,48,49^ Although there are multiple retrospective evaluations of ML models, there remain few prospective evaluations and even fewer prospective comparisons of clinician and ML predictions.^42,43,46,47,50,51^

We compared the prognostic performance of medical oncologists using an SQ with a supervised model trained to predict the risk of 3-month mortality. A motivator for this analysis was to improve the acceptability of ML for broader scale use in the electronic health record (EHR) and lay the groundwork to potentially increase prognostic confidence, improving discussions on goals of care between patients, families, and clinicians. This pilot study and the consequent evaluation were steps taken to validate the mortality prediction model and facilitate its acceptability among oncologists before integration in our EHR at the City of Hope National Medical Center.

## Methods

### Setting

This study was a comparison between predictions made by medical oncologists (57 physicians and 17 advanced practice clinicians) and their advanced practice clinicians at the City of Hope academic center and limited community sites and by a custom model running silently (ie, invisible to clinicians) for 20 months. Institutional review board approval was provided, along with a waiver of consent, by the City of Hope. The research needed a waiver of consent because the investigator does not have a reasonable opportunity to obtain consent and the risk inflicting psychological, social, or other harm by contacting participants is greater than the risk of the study procedures. The Standards for Reporting of Diagnostic Accuracy (STARD) reporting guideline was followed.^52^

### Background

#### 3-Month Surprise Question

Medical oncologists at City of Hope, a National Cancer Institute–designated cancer center, are adherent in use of a software decision support pathway tool capturing each episode of a patient’s systemic therapy.^53^ In a quaternary, highly specialized center for cancer care seeing many patients with advanced cancer, the oncologists believed a 3-month prognostic SQ would be meaningful to introduce in pathways as a trigger for goals-of-care discussions with patients regarding the benefits and burdens of additional therapy. We incorporated a mandatory 3-month surprise question (3MSQ) within the pathway tool for all patients with metastatic solid tumors in December 2019, worded “Would you be surprised if this patient were to die within the next 3 months?” The answer choices are yes or no.

#### The Machine Learning Model

We trained a gradient-boosted trees binary classifier (via the XGBoost library)^54^ with observations from 28 484 deceased and alive patients and 493 features from demographic characteristics, laboratory test results, flowsheets, and diagnoses collected from the EHR between January 1, 2013, and April 24, 2019. In training and retrospective evaluation, we considered 1 observation per patient. For inclusion in training and evaluation sets, patients needed to have at least 2 encounters as a minimal amount of data; living patients needed a completed visit documented in the EHR at least 1 year postprediction date to avoid observations with potential missing death information. To limit the risk of data leakage, we picked dates of prediction to exclude encounters within 7 days of death. We also avoided overrepresenting observations with prediction dates within 30 days of death to not train the model with a disproportionate number of near-term deceased patients. We extracted hand-crafted features from time series of laboratory test results and flowsheet data in the 180-day temporal window preceding each prediction. We imputed missing values for features only in obvious cases because tree-based classifiers can handle missing data. Clinical variables used by the model, including age, sex, race, and body mass index, and other features extracted from laboratory test and flowsheet time series, are listed in eTable 1 in the Supplement. The features associated with the diagnoses consisted of aggregations of publicly available word2vec embeddings^55^ of the *International Classification of Diseases, Ninth Revision* codes. A portion of the observations was used for retrospective evaluation based on a temporal split at a single time point to mimic deployment in the real world, in which past observations are used to train a model to predict in the present.

After hyperparameter tuning via cross-validation and retrospective evaluation, we retrained a version of the 90-day mortality model including the evaluation set and deployed it in a silent prospective pilot, inclusive of all City of Hope patients with accessible EHR data. The resulting model consisted of an ensemble of 357 decision trees with a maximum depth of 6. Since October 2019, the model made batches of predictions from observations automatically queried once a day from our enterprise data warehouse. The presence of new results from laboratory tests noted in eTable 2 in the Supplement triggered a prediction.

### Study Population

We identified all medical oncologist and advanced practice clinician (oncologist) 3MSQ answers entered into our pathway decision support tool when any new regimen was ordered for metastatic disease between December 6, 2019, and August 6, 2021, from our enterprise data warehouse. After excluding prognostications not associated with outpatient visits and those entered on or after a death date, we defined a cohort inclusive of oncologist prognostications with a model prediction for the same patient within the preceding 30 days. The cohort consisted of 3099 predictions from both the model and oncologists for 2041 patients with advanced cancer. The EHR data in the community network were not yet fully accessible to the model, which limited predictions for the comparison period. Most 3MSQs were associated with encounters at the academic center (2972 [95.9%]) vs community satellites (127 [4.1%]) (Table 1). Patient race and ethnicity information was self-reported as part of routine intake and collected from the enterprise data warehouse.

**Table 1.** Patients With Metastatic Solid Tumors Subject to the 3-Month Surprise Question Prognostications and Model Predictions

Variable	Cohort, No. (%)
Encounters/prognostications	3099
Patients	2041
Medical oncologists and advanced practice clinicians	74
Prognostication count per oncologist, mean (range)	41.9 (1-245)
Days between appointment and prognostication, median (SD)	2 (15.5)
Sex	
Male	770 (37.7)
Female	1271 (62.3)
Age, median (range), y^a^	62.6 (18-96)
Disease group	
Breast	482 (23.6)
Gastrointestinal	629 (30.8)
Genitourinary	280 (13.7)
Lung	378 (18.5)
Rare	272 (13.3)
Race	
American Indian/Alaska Native	12 (0.6)
Asian	479 (23.5)
Black/African American	102 (5.0)
White	1322 (64.8)
Other/unknown^b^	126 (6.2)
Ethnicity	
Hispanic or Latino	488 (23.9)
Not Hispanic or Latino	1498 (73.4)
Unknown/declined to answer	55 (2.7)

^a^ Age is reported at the encounter level.

^b^ Other includes Native Hawaiian and other Pacific Islander (9 [0.4%]), as well as other races not discretely captured within the electronic health record.

### Study Design

This prognostic study compared performance of 3-month mortality predictions made by oncologists and a model for patients with metastatic solid tumors. Oncologists’ predictions were made from December 6, 2019, to August 6, 2021, as answers to a 3MSQ. The predictions were paired with the closest model prediction within the preceding 30 days. For the entire study, oncologists and the model were blinded to the predictions of the other. The primary outcome was an in-depth comparison between the performance of oncologists and our model in predicting 3-month mortality for a population of patients with metastatic solid tumors seen in outpatient clinics.

### Statistical Analysis

The cohort included 3099 pairs of predictions for 2041 unique patients (Table 1). A prediction from the model comprises a score between 0 and 1 (a value close to 1 indicates high mortality risk). In contrast, oncologist predictions are binary: yes or no. To compare predictions among the model and oncologists, we set a threshold to convert risk scores into decisions (eg, flag patients scoring >0.5 as at risk of 3-month mortality). We set the decision threshold to match model sensitivity to that of the oncologists. Therefore, we compared oncologists and the model on positive predictive value (PPV or precision), which is the ratio of correct predictions over the total number of predictions made. The PPV depends on prevalence; thus, we also included the PPV-to-prevalence ratio to facilitate understanding of the results. In a scenario of pure random guessing, the PPV-to-prevalence ratio asymptotically converges to 1. We computed 95% CIs of the metrics via bootstrapping. For comparisons within disease groups, we computed 95% CIs of the difference between the PPV of the model and clinicians because 95% CIs of the PPV overlapped. A 95% CI of the difference above 0 means the model outperforms oncologists with statistical significance. Moreover, we characterized performance through sensitivity (or recall), specificity, and median lead days. We defined lead days as the number of days between a correct mortality prediction and the date of death. For the model, we also computed the area under the receiver operating characteristic curve (AUROC) and area under the precision-recall curve. The results for oncologists and the model are summarized in Table 2 with stratified evaluations over disease groups and changes in systemic therapy in Table 3. We also evaluated performance over a larger cohort of 3MSQs including answers without a model prediction (eMethods and eTable 3 in the Supplement).

**Table 2.** Performance of Oncologists Answering a 3-Month Surprise Question Compared With the 90-Day Mortality Prediction Model

Variable	Oncologists	ML model	Oncologist-ML model concordant decisions
No.	3099	3099	3099
Prevalence (90-d mortality), %	15.2	14.4	15.2
Area under the receiver operating characteristic curve, %	59.8 (57.7-62.0)	81.2 (79.1-83.3)	55.7 (54.2-57.3)
Area under the precision-recall curve, %	NA	46.2 (41.4-51.3)	NA
PPV (precision)	34.8 (30.1-39.5)	60.0 (53.6-66.3)	68.6 (58.2-78.4)
Sensitivity (recall), %	29.7 (25.6-33.8)	29.5 (25.4-34.0)	12.5 (9.6-15.6)
Specificity, %	90.0 (88.9-91.2)	96.7 (96.0-97.3)	99.0 (98.6-99.4)
PPV-to-prevalence ratio	2.3 (2.0-2.6)	4.2 (3.7-4.7)	4.5 (3.8-5.2)
Negative predictive value, %	87.7 (86.4-88.9)	89.1 (87.9-90.2)	86.3 (85.1-87.5)
Median lead days	37.5 (31.5-45.0)	28.5 (25.0-36.0)	30.0 (20.5-32.5)

Abbreviations: ML, machine learning; NA, not applicable; PPV, positive predictive value.

**Table 3.** Performance of Medical Oncologists With a 3-Month Surprise Question Compared With an ML Model With Stratification by Disease Groups and Presence of Systemic Therapy Changes

**Disease group**	**No.**	**90-d Mortality, %**	**AUROC**	**PPV to prevalence**	**Sensitivity, %**	**PPV, %**	**PPV difference, 95% CI, %** ^a^
**All disease groups**							
Oncologists	3099	15.2	NC	2.3 (2.0 to 2.6)	29.7	34.8	18.5 to 31.9
ML model		14.4	81.2 (79.1 to 83.3)	NC	29.5	60.0	
**Breast**							
Oncologists	697	10.3	NC	3.5 (2.7 to 4.6)	37.5	36.5	1.7 to 32.5
Model		9.9	87.3 (83.0 to 91.1)	NC	36.2	53.2	
**Gastrointestinal**							
Oncologists	937	15.4	NC	2.1 (1.8 to 2.4)	52.1	32.5	4.1 to 18.5
ML model		14.4	81 (76.8 to 85.0)	NC	52.6	43.8	
**Genitourinary (including gynecologic)**							
Oncologists	376	12	NC	2.7 (1.4 to 4.2)	20	32.1	−15.7 to 35.6
ML model		11.2	85 (78.8 to 90.5)	NC	19	42.1	
**Lung**							
Oncologists	639	22.8	NC	2 (1.2 to 2.9)	9.6	46.7	−10.6 to 43.4
ML model		21.6	77.7 (73.2 to 82.2)	NC	10.1	63.6	
**Rare**							
Oncologists	450	14.4	NC	2.7 (1.7 to 3.8)	23.1	38.5	−1.3 to 45.3
ML model		14	76.4 (69.3 to 82.8)	NC	22.2	60.9	
**Patients with no change in therapy**							
Oncologists	1333	13.4	NC	2.5 (2.0 to 3.0)	26.8	33.1	5.9 to 26.0
ML model		13.2	80.1 (76.6 to 83.6)	NC	27.3	49	
**Patients with changes in therapy**							
Oncologists	1766	16.6	NC	2.2 (1.8 to 2.5)	31.4	35.8	19.7 to 37.2
ML model		15.4	82.4 (79.7 to 85.0)	NC	31.7	64.2	

Abbreviations: AUROC, area under the receiving operator characteristic curve; ML, machine learning; NC, not calculated; PPV, positive predictive value.

^a^ If the 95% CI of the precision difference does not include 0, the precision of the model is statistically significantly better than that of the oncologists.

## Results

We evaluated 3099 pairs of 3-month mortality predictions by oncologists and the model for 2041 patients (1271 [62.3%] women; 770 [37.7%] men) with a median age of 62.6 (range, 18-96) years at the time of oncologist prediction. The median lag between a 3MSQ answer and a model prediction was 3 days, with 75% of model predictions made within 8 days from the corresponding answer. We compared model and oncologist performance by setting a decision threshold so that model sensitivity matched the 30% sensitivity of the oncologists. Results showed that the model outperformed oncologists in aggregate (PPV, 60.0%; 95% CI, 53.6%-66.3% vs 34.8%; 95% CI, 30.1%-39.5%; *P* < .001) (Table 2) and within breast (PPV difference, 16.7%; 95% CI, 1.7%-32.5%; *P* = .03) and gastrointestinal (PPV difference, 11.3%; 95% CI, 4.1%-18.5%; *P* = .002) disease subgroups (ie, 95% CI PPV difference >0) (Table 3). The PPV difference was not statistically significant within genitourinary (including gynecologic), lung, and rare cancer groups. For concordant oncologist-model predictions, the PPV of the oncologists increased from 34.8% (95% CI, 30.1%-39.5%) to 68.6% (95% CI, 58.2%-78.4%), with a decrease in sensitivity to 12.5% (Table 2).

**Figure 1.**
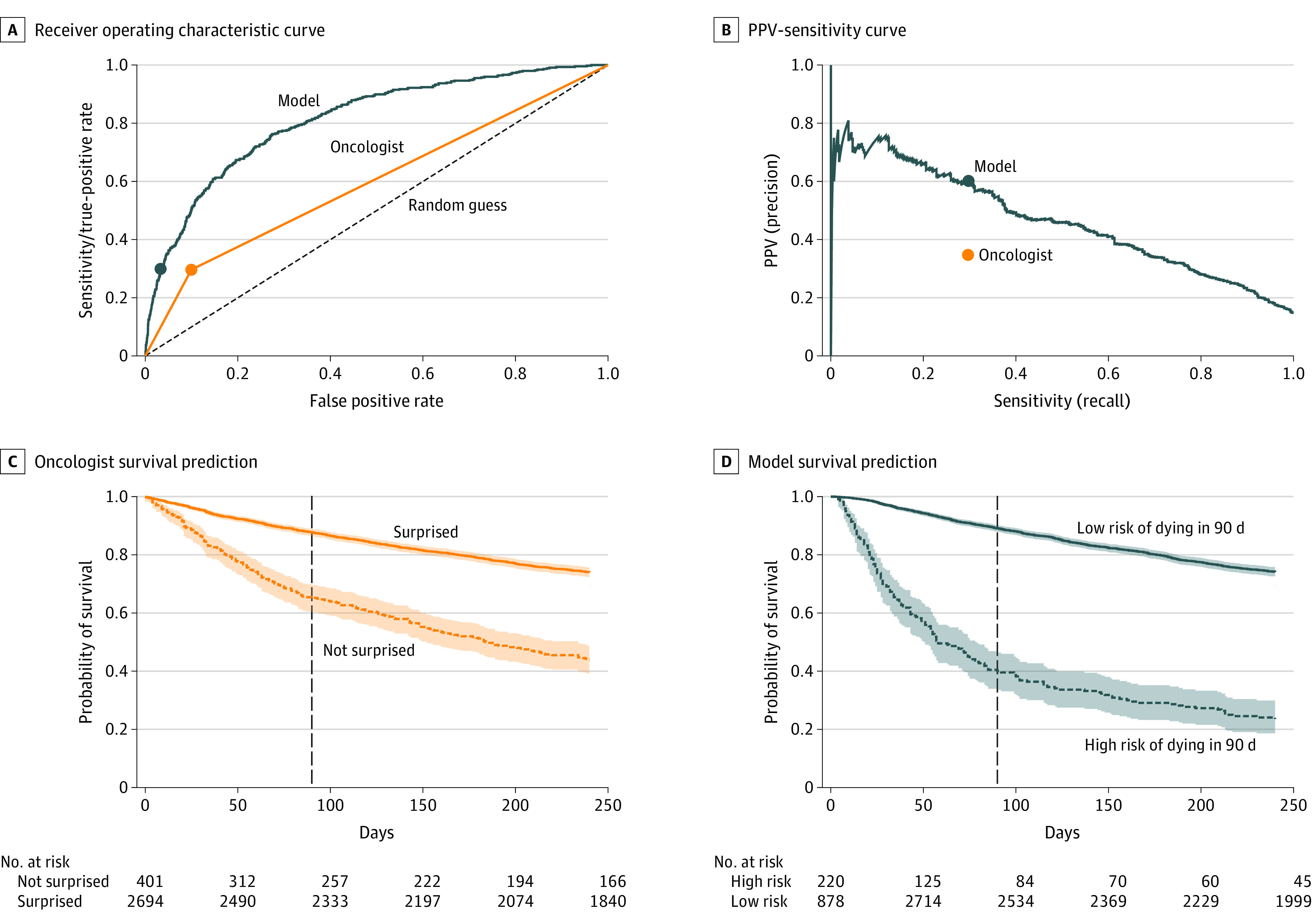
Receiver Operating Characteristic Curve (ROC), Precision Recall Curve (PRC), and Survival Curves for the Model and Oncologists Receiver operating characteristic curve (A) and positive predictive value (PPV)–sensitivity or PRC (B) for the machine learning model (model) vs oncologists. The model area under the ROC curve was 81%; area under the PRC, 50%; and prevalence, 14.4%. In survival plots for oncologists (C) and the model (D), the continuous curve was associated with a predicted low risk of death and, in the ideal case, would be horizontal for the first 3 months.

**Figure 2.**
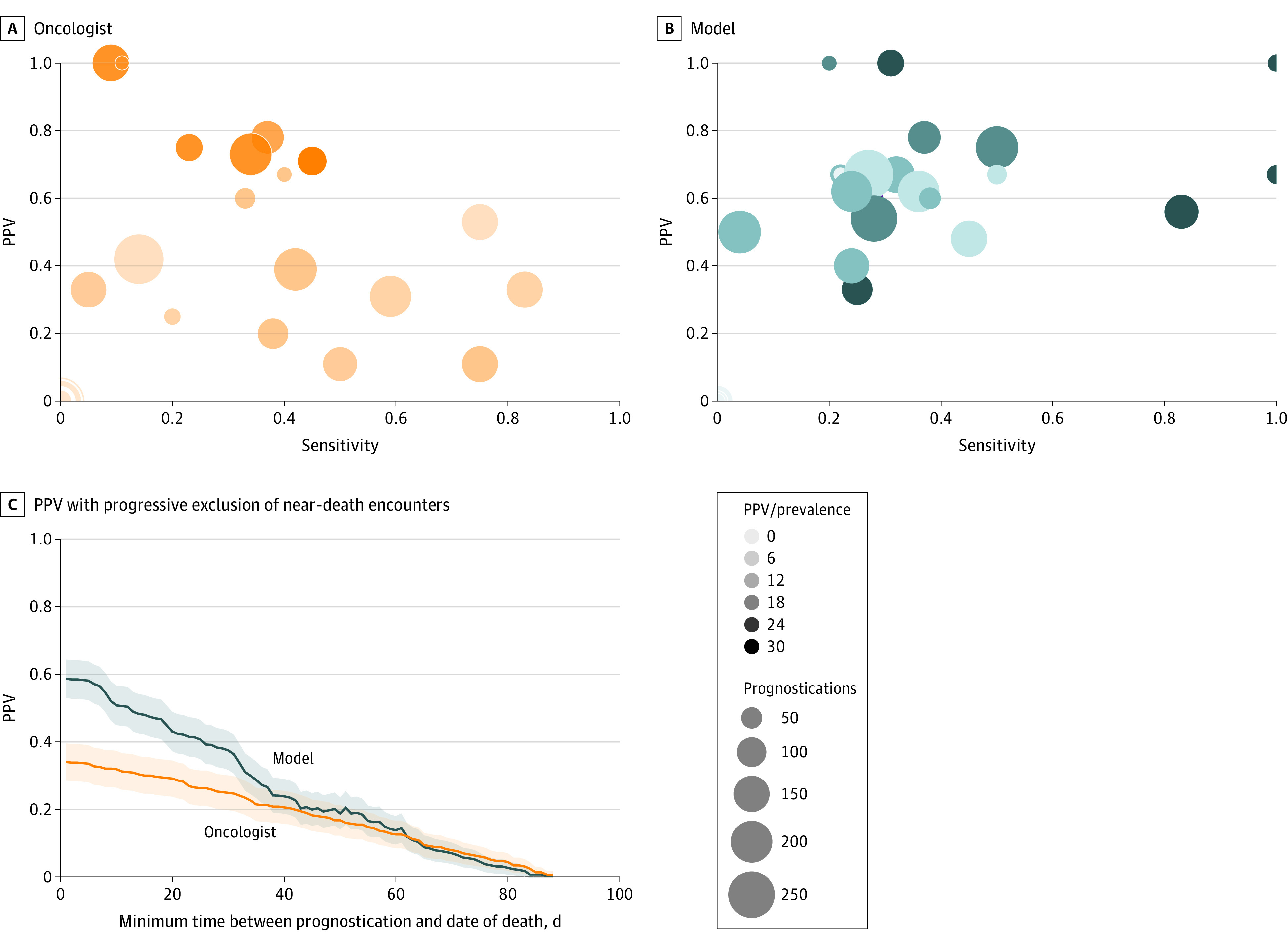
Positive Predictive Value (PPV) and Sensitivity Scatterplots in PPV Plots With Exclusion of Near-Death Encounters A, For oncologists, the PPV was 34.8% and sensitivity was 29.7%. B, For the machine learning model, PPV was 60.0% and sensitivity was 29.5%. Each dot in the scatterplots corresponds to the prognostications of an oncologist (A) and to associated model predictions (B). The size of a dot is proportional to the prediction count, and the hue represents the ratio of PPV over prevalence (darker color indicates better performance). C, Comparison of PPV for the model and oncologists with progressive exclusion of near-death encounters.

Figure 1A and B display ROC and PPV sensitivity curves for the model compared with sensitivity, false-positive rates, and PPV sensitivity for the oncologists. The model AUROC was 81.2% (95% CI, 79.1%-83.3%). The AUROC for the oncologists was 59.8% (95% CI, 57.7%-62.0%). The model operating at the same sensitivity level of the oncologists achieved lower false-positive rates and higher PPVs. Survival curves for oncologists (Figure 1C) and the model (Figure 1D) further show the better discriminative ability of the latter. Scatterplots show PPV and sensitivity for individual oncologists with at least 20 prognostications (Figure 2A) and associated model predictions (Figure 2B). Dots in the origin indicate instances of exclusively incorrect predictions. The plots show higher consistency of the model predictions (ie, the points are less scattered).

Figure 2C displays how the PPV for the model and oncologists varies with progressive exclusion of near-death encounters. For instance, with no encounter within 36 days from death, PPVs of the model and oncologists would be comparable. eFigure 1 in the Supplement shows the most predictive model features over the cohort. eFigure 2 in the Supplement shows that most oncologists predicted risk of death for their patients (ie, answered surprised for a fraction of instances smaller than the related prevalence). This finding could be interpreted as a conservative approach to privilege PPV rather than sensitivity.

### Stratification by Changes in Therapy

Because of changes in therapy, 708 patients had multiple (up to 6) SQs answered by clinicians, encompassing 1766 predictions (57.0%). We split the cohort between patients with 1 SQ and patients with multiple SQs and included all SQ answers in our analysis. The difference between the PPV of the model and oncologists was larger for the subpopulation with changes in therapy (PPV difference, 28.4%; 95% CI, 19.7%-37.2%; *P* < .001 vs PPV difference, 15.9%; 95% CI, 5.9%-26.0%; *P* = .002) (Table 3). We also observed that, for this subpopulation, the oncologists changed predictions for only 31 patients, but the correct prognosis should have changed for 155 patients.

### COVID-19

COVID-19 was associated with population shifts in our organization, especially between March and August 2020 when visits were deferred or switched to telehealth if clinically appropriate. The AUROC of the model over the entire patient population (beyond the metastatic solid tumor cohort in this study) decreased during late spring 2020 but then increased in the following summer months. However, in this metastatic cohort, we observed fluctuations in the monthly performance of the model and oncologists without clear temporal patterns, perhaps owing to the homogeneity of the cohort over time. eFigure 3 in the Supplement shows a quarterly comparison of PPV and prevalence between the model and oncologists.

## Discussion

In this prognostic study, we compared the performance of medical oncologists and their advanced practice clinicians with an ML model to predict 3-month mortality for a cohort of patients with metastatic solid tumors at City of Hope. At a sensitivity of 30% (matching the oncologists’ performance), the model outperformed the oncologists in PPV (60.0% vs 34.8%; *P* < .001) to predict 3-month mortality with 15% prevalence. Stated differently, the model matched the sensitivity of the oncologists but flagged nearly half of the instances (7.7% vs 14.1%). Looking forward, we anticipate tuning the model threshold to increase sensitivity or PPV depending on the clinical application but, more importantly, augmenting oncologists’ prognostic capability while tempering overestimation of prognosis.

The model outperformed oncologists across each disease group, with a statistically significant margin for the 2 largest groups: breast (*P* = .03) and gastrointestinal (*P* = .002) cancer. The model AUROC was consistent across all groups (Table 3). To facilitate performance comparison across subpopulations with different prevalence, we evaluated the PPV-to-prevalence ratio. The 95% CIs for the oncologist PPV-to-prevalence ratio were above 1 across all disease groups (Table 3), indicating that oncologist performance was significantly better than random guessing.

In an SQ systematic review by White et al,^7^ clinicians with 12-month and 7-day (1 study) SQs in oncology populations achieved an AUROC in the 66% to 82% range, with a 75% mean.^40,50,56^ None of the studies limited the population to patients with metastatic solid tumors, and few had oncologists prognosticating. The study most similar to ours was by Vick et al,^40^ wherein 81 oncologists answered 4617 twelve-month SQs for a mixed population of patients with metastatic and nonmetastatic cancer, achieving a 74% AUROC. The oncologists in our study achieved a 59.8% (95% CI, 57.7%-62.0%) AUROC, but for a population of patients exclusively with metastatic disease. We believe that for a mixed population, oncologists would have achieved a higher AUROC. The AUROC for evaluation of binary (yes vs no) decisions has limitations because the ROC curve is defined only by 3 points (Figure 1A). After computing the PPV-to-prevalence ratio for the aforementioned oncologic studies,^7,28,31,32,40^ we observed that the performance of our clinicians was within range (eTable 4 in the Supplement).

In addition, we compared the model and oncologists’ PPVs after progressively excluding predictions associated with near-death encounters. Figure 2C shows that PPVs for the model and oncologists become similar after excluding predictions that are 35 days from the death date. In other words, the model gains in performance over oncologists on near-death cases, which is supported in a study in which higher performance of mortality prediction models occurred in near-death encounters.^57^ The consequent higher fraction of correctly predicted near-term deaths results in a lower median of lead days for the model compared with the oncologists.

### Contributions

To our knowledge, ours is the first reported prospective comparison of a model with oncology clinician prognostications for patients with metastatic cancer.^42^ The most similar prospective study, conducted by Manz et al,^46^ reported that a model trained on EHR data from a population of patients with hematologic and oncologic disease could be clinically useful and outperformed Eastern Cooperative Oncology Group and Elixhauser prognostic indices.

We provided insights on how our model outperformed oncologists by stratifying the cohort according to 3 criteria: (1) exclusion of patients near death, (2) different disease group subpopulations, and (3) presence and absence of therapy changes. Besides improved performance in predicting near-term mortality, the model was more consistent in predicting 3-month mortality compared with the widely varying performance seen for individual oncologists (Figure 2B), which is not readily apparent when looking at the oncologists in aggregate (Figure 1B).

We show the PPV-to-prevalence ratio as a potentially helpful metric for comparing the performance of binary predictions (surprised or not surprised) across subpopulations with different prevalence. In a situation of random guessing, the PPV-to-prevalence ratio would tend to 1. Several publications evaluating SQs and even clinical ML did not report prevalence with PPV by omitting it, leaving its extrapolation to the reader or presenting results close to random guessing (ie, with PPV close to prevalence) as instances of good performance.^27,28,29,49^

### Limitations

This study had limitations. The cohort (n = 3099) excluded most prognostications made at clinical network sites because the model was designed to work with laboratory tests performed at the academic cancer center but ordered infrequently at clinical network sites. Model upgrades will enhance coverage of patients receiving care at clinical network sites.

Communication of external deaths may be delayed by up to 6 months, with consequent underestimated prevalence and adjustments of performance results. For instance, the 3-month mortality rate for the cohort increased almost 1% in the span of 1 month. We observed a slow widening of the PPV difference between model and oncologists as death information was updated.

This blinded study evaluated the performance of the model and clinicians separately. To estimate the impact of model predictions in clinical practice, we need to evaluate how clinicians prognosticate once given the model predictions. Even a perfect model would have no benefit if it were not trusted by clinicians.

## Conclusions

In this prognostic study, we noted that an ML model trained with EHR data outperformed clinicians in predicting 3-month mortality for a cohort of patients with metastatic solid tumors, with a 95% CI of 18.5%-31.9% for the difference in PPVs at 30% sensitivity. The model was trained on a relatively small population (n = 28 484) and yet achieved an 81.2% AUROC on the cohort. This finding could encourage other centers to develop custom models based on internally available patient data. This study was a step to validate the model before integration in the EHR of our organization.^58^ Further evaluation is ongoing to study the performance of oncologists given model prognostications and whether use of the model improves prognostic confidence, patient engagement, and use of resources at the end of life.

## Notes

Supplement. **eTable 1.** Variables and Features Used by the Machine Learning Model **eMethods.** Oncologist Performance Over the Whole Clinical Pathway Cohort **eTable 2.** The Cohort of Patients with Metastatic Solid Tumors Subject to the 3-Month Surprise Question **eTable 3.** Performance of Oncologists Answering a 3-Month Surprise Question at COH **eFigure 1.** Most Predictive Features for the Machine Learning Model **eFigure 2.** “Mental Threshold” Distribution of Oncologists **eFigure 3.** Quarterly Comparison of Positive Predictive Value to Prevalence Ratio **eTable 4.** Performance of a Surprise Question in Oncology Across Different Studies
eReferences
