# Supplementary material for: Prospective Comparison of Medical Oncologists and a Machine Learning Model to Predict 3-Month Mortality in Patients With Metastatic Solid Tumors
Source: JAMA Netw Open. 2022 May 31;5(5):e2214514. doi: 10.1001/jamanetworkopen.2022.14514 (PMC9157269; doi:10.1001/jamanetworkopen.2022.14514)
Supplement: Supplement. — eTable 1. Variables and Features Used by the Machine Learning Model eMethods. Oncologist Performance Over the Whole Clinical Pathway Cohort eTable 2. The Cohort of Patients with Metastatic Solid Tumors Subject to the 3-Month Surprise Question eTable 3. Performance of Oncologists Answering a 3-Month Surprise Question at COH eFigure 1. Most Predictive Features for the Machine Learning Model eFigure 2. “Mental Threshold” Distribution of Oncologists eFigure 3. Quarterly Comparison of Positive Predictive Value to Prevalence Ratio eTable 4. Performance of a Surprise Question in Oncology Across Different Studies eReferences [file jamanetwopen-e2214514-s001.pdf]

Supplementary Online Content

Zachariah FJ, Rossi LA, Roberts LM, Bosserman LD. Prospective comparison of medical oncologists and a machine learning model to predict 3-month mortality in patients with metastatic solid tumors. *JAMA Netw Open*. 2022;5(5):e2214514.
doi:10.1001/jamanetworkopen.2022.14514

eTable 1. Variables and Features Used by the Machine Learning Model

eMethods. Oncologist Performance Over the Whole Clinical Pathway Cohort

eTable 2. The Cohort of Patients with Metastatic Solid Tumors Subject to the 3-Month Surprise Question

eTable 3. Performance of Oncologists Answering a 3-Month Surprise Question at COH

eFigure 1. Most Predictive Features for the Machine Learning Model

eFigure 2. "Mental Threshold" Distribution of Oncologists

eFigure 3. Quarterly Comparison of Positive Predictive Value to Prevalence Ratio

eTable 4. Performance of a Surprise Question in Oncology Across Different Studies

eReferences

This supplementary material has been provided by the authors to give readers additional information about their work.

© 2022 Zachariah FJ et al. *JAMA Network Open.*

eTable 1. Variables and Features Used by the Machine Learning Model

category	clinical variable	feature
Laboratory Tests	Lymphocite%, Albumin (Blood), Calcium (Blood), WBC, LDH (Blood), Hemoglobin (Blood), Platelet Count, Alkaline Phosphatase, Creatinine (Blood), Bilirubin (Blood), RBC, B12 (Serum), Segmented Neutrophil%	count, median, minimum, maximum, standard deviation, slope, intercept, early/late difference to lower/upper reference, % normal results, % very abnormal results % lab late night orders
Flowsheet	weight, BMI	count, median, minimum, maximum, standard deviation, slope, intercept, early/late difference to l/u ref.
Demographics	age, gender, race	value
Diagnoses	ICD-9 codes	embedding aggregations

© 2022 Zachariah FJ et al. *JAMA Network Open.*

eMethods. Oncologist Performance Over the Whole Clinical Pathway Cohort

The manuscript focuses on a cohort of paired predictions made by oncologists and a machine learning model for ambulatory patients, where the former ones were captured as responses to a 3-month surprise question (3MSQ) entered in a clinical pathway tool. A model's prediction was included in the cohort if it occurred between 0 and 30 days before the oncologist's prediction for the same patients. 2785 oncologist predictions, typically occurred at the satellite clinical networks, did not have a paired model prediction. The performance of the oncologist predictions for this larger clinical pathway cohort is included here for transparency around patient characteristics and completeness. The cohort described in the manuscript is a subset of this one. The demographics of the cohort are presented in eTable 2.

Performance across the entire clinical pathway cohort is summarized in eTable 3. The 95% confidence interval of the positive predictive value (PPV) to prevalence ratio for the oncologists is above one for the study cohort. Review of the clinical pathway cohort showed performance similar to the study cohort for the medical oncologists, wherein oncologists achieved 30.2% PPV and 26% sensitivity on 5864 predictions for 4070 unique patients, with a 12.5% 90-day mortality rate.

© 2022 Zachariah FJ et al. *JAMA Network Open.*

eTable 2. The Cohort of Patients with Metastatic Solid Tumors Subject to the 3-Month Surprise Question

	Clinical Pathway Cohort
**Encounters/Prognostications**	5864
**Patients**	4070
**Medical Oncologists & Advanced Practice Providers**	88
**Mean Prognostication Count Per Oncologist (range)**	66.6 (1 – 327)
**Mean Days Between Appointment and Prognostication**	5
**Gender**	
Male	1595 (39.2%)
Female	2473 (60.8%)
**Median Age (range)[a]**	63.7 (18 – 98)
**Disease Group**	
Breast	886 (21.8%)
Gastrointestinal	1215 (29.9%)
Genitourinary	766 (18.8%)
Lung	697 (17.1%)
Rare	506 (12.4%)
**Race**	
American Indian/Alaska Native	21 (0.6%)
Asian	735 (18.1%)
Black/African American	218 (5.4%)
White	2690 (66.1%)
Other/Unknown	400 (9.8%)
**Ethnicity**	
Hispanic or Latino	980 (24.1%)
Not Hispanic or Latino	2852 (70.1%)
Unknown / Decline to Answer	236 (5.6%)

[a]Age is reported at the encounter level.

© 2022 Zachariah FJ et al. *JAMA Network Open.*

eTable 3. Performance of Oncologists Answering a 3-Month Surprise Question at COH

	Clinical Pathway Cohort
**N**	5864
**Prevalence (90-day Mortality) %**	12.5
**Area Under the Receiver Operating Characteristic %**	58.7 (57.1-60.3)
**Positive Predictive Value (PPV, Precision)**	30.2 (26.6-33.8)
**Sensitivity (Recall) %**	26 (22.8-29.1)
**Specificity %**	91.4 (90.7-92.2)
**PPV To Prevalence Ratio**	2.4 (2.2-2.7)
**Negative Predictive Value %**	89.6 (88.8-90.4)
**Median Lead Days**	36.5 (32-44)

© 2022 Zachariah FJ et al. *JAMA Network Open.*

eFigure 1. Most Predictive Features for the Machine Learning Model

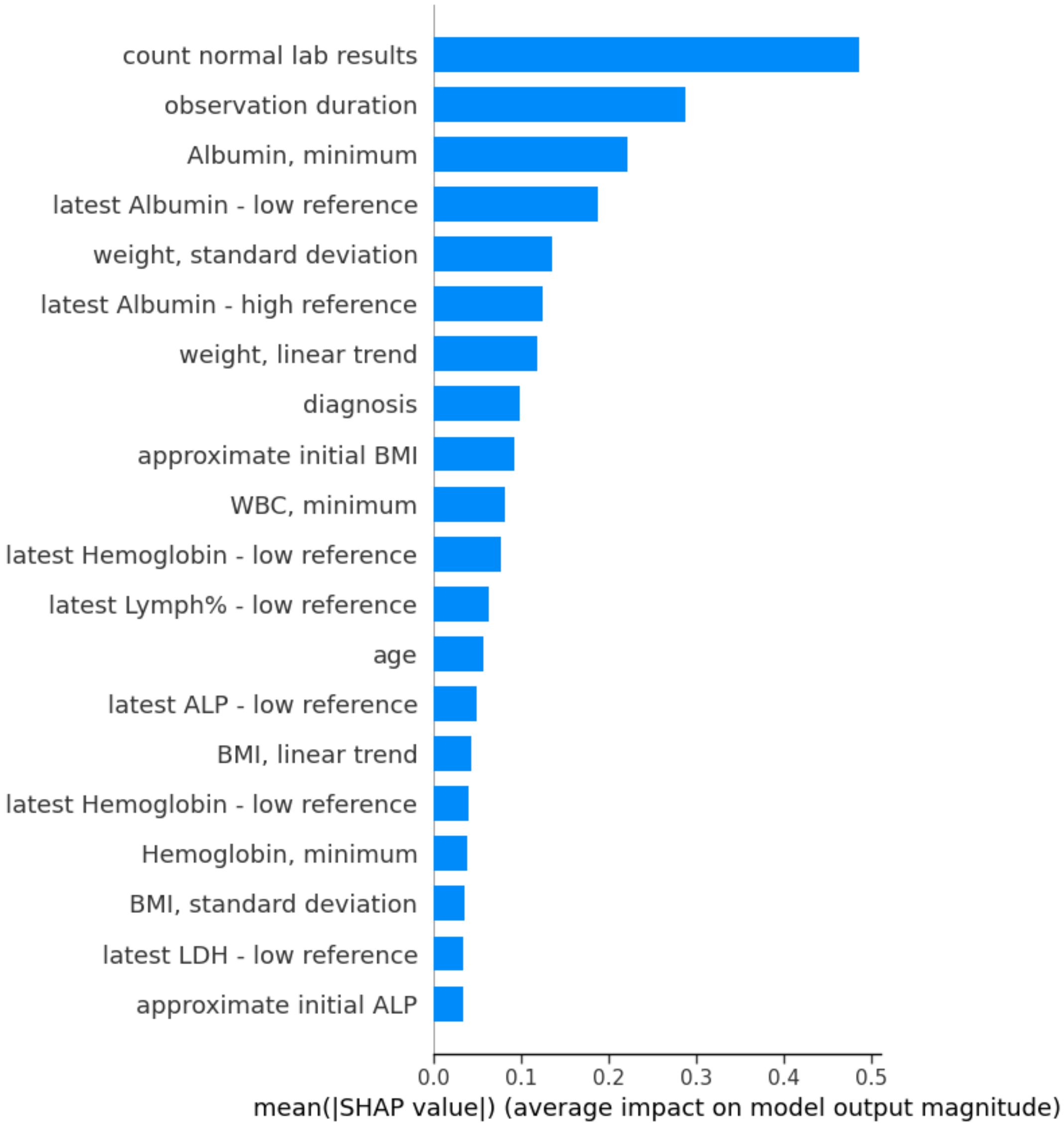

The most predictive features for the 90-day mortality model used in the study. E.g. “latest Albumin – low reference” is the difference between the most recent value of Albumin Level lab test and the related low reference. Lab, weight and BMI features are extracted from a 6-month time window. “Approximate initial BMI” is the intercept of the linear interpolation of the BMI time series. BMI: body mass index, WBC: white blood cell, LDH: lactate dehydrogenase, ALP: alkaline phosphatase.

© 2022 Zachariah FJ et al. *JAMA Network Open.*

eFigure 2. “Mental Threshold” Distribution of Oncologists

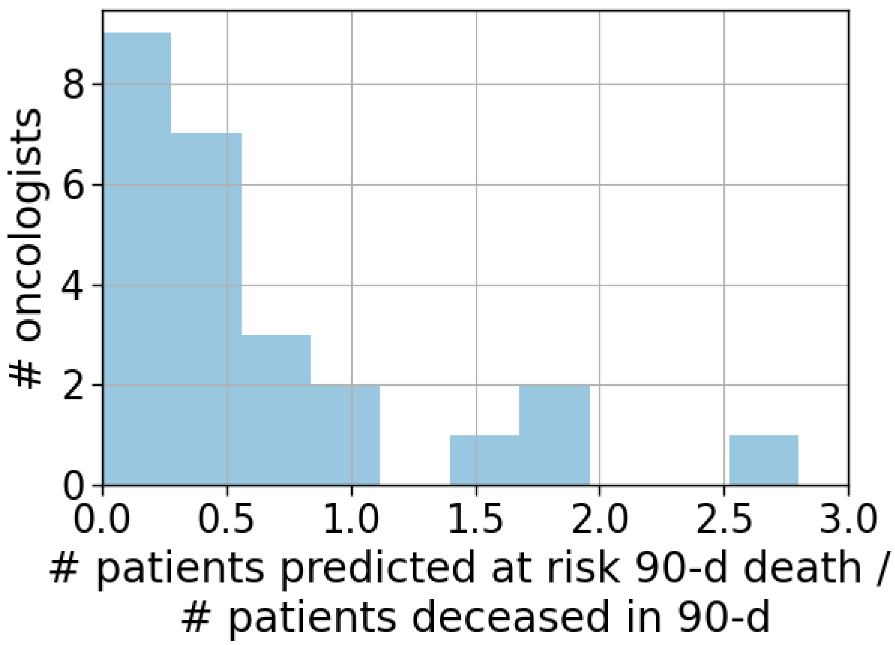

The bar chart displays the distribution of the oncologist’s “mental threshold”. This has been restricted to oncologists with at least 20 prognostications. Oncologists with fewer than 20 predictions have been aggregated as one single instance. For example, for 6 oncologists the ratio between the count of patients they predicted as at risk of death in 90 days and the count of patients who died in 90 days was roughly between 0.28 and 0.56. In other words, most oncologists in this study forecasted more conservatively with respect to the 90-day mortality rate (i.e. the above ratio is < 1). In the trade-off between positive predictive value (PPV) and sensitivity, most clinicians in our study seemed to prioritize PPV.

© 2022 Zachariah FJ et al. *JAMA Network Open.*

eFigure 3. Quarterly Comparison of Positive Predictive Value to Prevalence Ratio

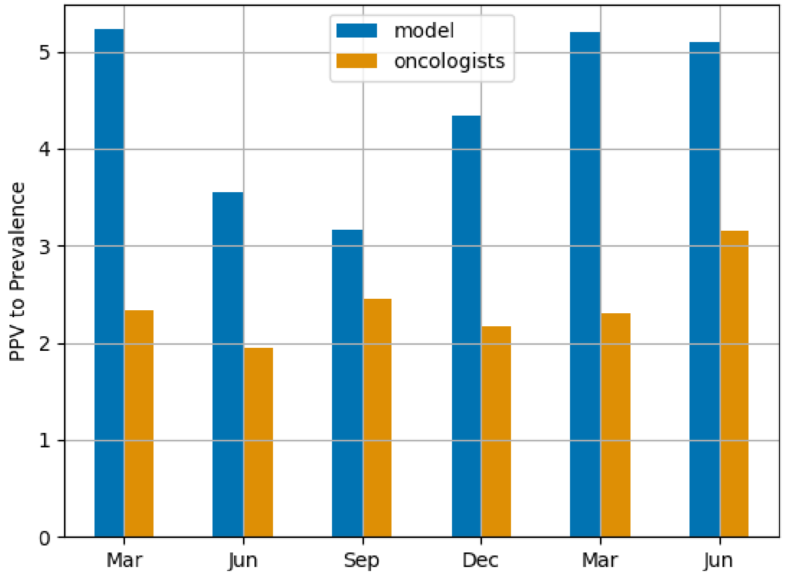

© 2022 Zachariah FJ et al. *JAMA Network Open.*

eTable 4. Performance of a Surprise Question in Oncology Across Different Studies[1]

	Timeframe of Surprise Question	Cohort Size	Deaths	PPV	Prevalence	PPV to Prevalence
**Oncologists from** our **Study (Clinical Pathway Cohort)**	3 months	5864	733	30.2 (26.7-33.8)	12.5	2.4 (2.2-2.7)
**Oncologists from** our **Study (Manuscript Cohort)**	3 months	3099	471	34.8 (30-39.5)	15.2	2.3 (2-2.6)
**Hamano *et al.*, 2015**[2]	7 days	2361	333	30.3	14.1	2.13
**Halbe *et al.*, 2015**[3]	12 months	651	108	51.1	16.59	3.08
**Lefkowits *et al.* 2015**[4]	12 months	263	54	--	--	NA
**Moroni *et al.* 2014**[5]	12 months	231	104	69	45.02	1.53
**Vick *et al.* 2015**[6]	12 months	4617		47	13.88	3.39

© 2022 Zachariah FJ et al. *JAMA Network Open.*

eReferences

1. White N, Kupeli N, Vickerstaff V, Stone P. How accurate is the 'Surprise Question' at identifying patients at the end of life? A systematic review and meta-analysis. *BMC Med.* 2017;15(1):139-139.
2. Hamano J, Morita T, Inoue S, et al. Surprise Questions for Survival Prediction in Patients With Advanced Cancer: A Multicenter Prospective Cohort Study. *Oncologist.* 2015;20(7):839-844.
3. Halbe L, Gerlach C, Hess G, et al. "Would I be surprised if this patient died in the next year?" - Prognostic significance of the "Surprise" Question in a university hematology and oncology outpatients clinic. Jahrestagung der Deutschen, Osterreichischen und Schweizerischen Gesellschaften fur Hamatologie und Medizinische Onkologie. Basel: S. Karger AG; 2015.
4. Lefkowits C, Chandler C, Sukumvanich P, et al. Validation of the "surprise question" in gynecologic oncology: Comparing physicians, advanced practice providers and nurses. *Journal of Clinical Oncology.* 2015;33(29_suppl):151-151.
5. Moroni M, Zocchi D, Bolognesi D, et al. The 'surprise' question in advanced cancer patients: A prospective study among general practitioners. *Palliative medicine.* 2014;28(7):959-964.
6. Vick JB, Pertsch N, Hutchings M, et al. The utility of the surprise question in identifying patients most at risk of death. *Journal of Clinical Oncology.* 2015;33(29_suppl):8-8.

© 2022 Zachariah FJ et al. *JAMA Network Open.*
